# Insertion and deletion correcting DNA barcodes based on watermarks

**DOI:** 10.1186/s12859-015-0482-7

**Published:** 2015-02-18

**Authors:** David Kracht, Steffen Schober

**Affiliations:** Institute of Communications Engineering, Ulm University, Albert-Einstein-Allee 43, Ulm, 89081 Germany

**Keywords:** Next generation sequencing, Barcodes, Multiplexing, Edit distance, Watermark codes, Insertions, Deletions, Sequence embedding

## Abstract

**Background:**

Barcode multiplexing is a key strategy for sharing the rising capacity of next-generation sequencing devices: Synthetic DNA tags, called barcodes, are attached to natural DNA fragments within the library preparation procedure. Different libraries, can individually be labeled with barcodes for a joint sequencing procedure. A post-processing step is needed to sort the sequencing data according to their origin, utilizing these DNA labels. The final separation step is called demultiplexing and is mainly determined by the characteristics of the DNA code words used as labels.

Currently, we are facing two different strategies for barcoding: One is based on the Hamming distance, the other uses the edit metric to measure distances of code words. The theory of channel coding provides well-known code constructions for Hamming metric. They provide a large number of code words with variable lengths and maximal correction capability regarding substitution errors. However, some sequencing platforms are known to have exceptional high numbers of insertion or deletion errors. Barcodes based on the edit distance can take insertion and deletion errors into account in the decoding process. Unfortunately, there is no explicit code-construction known that gives optimal codes for edit metric.

**Results:**

In the present work we focus on an entirely different perspective to obtain DNA barcodes. We consider a concatenated code construction, producing so-called watermark codes, which were first proposed by Davey and Mackay, to communicate via binary channels with synchronization errors. We adapt and extend the concepts of watermark codes to use them for DNA sequencing. Moreover, we provide an exemplary set of barcodes that are experimentally compatible with common next-generation sequencing platforms. Finally, a realistic simulation scenario is use to evaluate the proposed codes to show that the watermark concept is suitable for DNA sequencing applications.

**Conclusion:**

Our adaption of watermark codes enables the construction of barcodes that are capable of correcting substitutions, insertion and deletion errors. The presented approach has the advantage of not needing any markers or technical sequences to recover the position of the barcode in the sequencing reads, which poses a significant restriction with other approaches.

**Electronic supplementary material:**

The online version of this article (doi:10.1186/s12859-015-0482-7) contains supplementary material, which is available to authorized users.

## Background

Due to the steadily increasing throughput on platforms for next-generation sequencing and dropping prices of commercially available devices, DNA sequencing becomes broadly accessible for researchers. Since the output of bases in each sequencing run has reached giga to tera orders within the last few years, strategies for efficiently sharing the sequencing capacity has become of particular interest. Multiplexed sequencing is a major key technique, that makes sequencing devices accessible in parallel: DNA samples from different experiments can be pooled into batches and sequenced in parallel in a single sequencing run. Before joining different samples it is mandatory to uniquely label the DNA fragments. DNA barcodes, artificially synthesized sequences of nucleic acids, are used as labels to tag the fragments and to separate the output of the sequencers according to the input samples.

The robustness of multiplexing in general relies on the properties of the used barcodes and how well they are adapted to the underlying sequencing protocol and platform. Essential experimental pre-processing steps, which are needed to prepare the DNA material can cause errors on the target genomic sequence and the barcodes as well. Physical and chemical sequence modifications, e.g. fragmentation, ligation, or copy procedures are known sources of such errors. These errors lead to cross-talk during demultiplexing, i.e., sequences from different batches can not clearly be distinguished, which is of course highly undesirable.

Different constructions for barcodes have been proposed, for example those of Hamady et al. [1] and Bystrykh [2] are based on Hamming codes [3] or the approach of Krishnan et al. [4] based on BCH codes [5], to name just a few. For short lengths it is even feasible to apply brute-force search techniques, e.g. Frank [6] or Mir et al. [7], where some of the resulting codes of the latter approach even reach fundamental bounds. The constructions mentioned so far are designed to correct substitution errors only. From a conceptional point of view all of them try to provide codes that maximize the so-called Hamming distance between the individual code words. The Hamming distance between a pair of sequences measure the minimal number of symbol-wise substitution that are needed to transform them into each other. But, some specific sequencing platforms are known to have exceptional high numbers of insertion and deletion errors as reported for Roche 454 Pyrosequencing [8], PacBio sequencers [9] or Ion Torrent PGM [10]. See [11-13] for a comparison of these sequencing techniques. Hence, especially for insertion and deletion prone devices one has to consider barcodes that are capable to correct indels.

Promising attempts to find barcodes that are robust to indels have been considered in [14,15] using the so-called *edit* or *Levenshtein* distance (see [16] for an overview). For calculating the distance between code words the Levenshtein distance takes insertion and deletion operations into account and is therefore better suited for applications where decoding based on Hamming distance fails. Unfortunately, there is no code-construction known that directly gives optimal codes in edit metric. Some greedy (later evolutionary) algorithms has been proposed in [17,18] to find sets of barcodes of moderate size with high minimal edit distance, additionally fulfilling biological constraints. However, a practical decoding step for the obtained barcodes has not been addressed in the mentioned papers. This was later done by [19], where it is stated that maximizing the edit distance for barcodes (within a sequence context) is a sub-optimal or even wrong strategy. The context of a code words, which is simply the sequence that contains the DNA tag plays an important role. Due to indels the exact boundaries of code words can not be correctly recovered. This leads to additional errors, if the sequence context was not included in the code construction. The DNA context at one end of a code word can be taken into account by using an adapted *Sequence-Levenshtein distance*, as proposed in [19].

In this manuscript, we provide an entire different perspective to obtain barcodes. We give codes based on concepts introduced by Davey and Mackay [20]. The original watermark approach is aimed to synchronize and decode a continuous stream of large binary data-blocks. In the domain of DNA codes we face additional constraints, for which the original concept is adapted. We finally give an exemplary set of barcodes and provide an in silico application, which shows that demultiplexing based on the watermark concept is applicable in the field of next-generation sequencing. Basic concepts of watermark coding has already been considered for data embedding in DNA [21,22], which is closely related to the barcoding approach for DNA sequencing. But, the transmission of biologically compatible sequences through an evolutionary channel (in living cells) is only slightly similar to the approach we consider in the present manuscript.

Note, that search approaches like [16,19] can be used to find better codes in terms of code rate and minimal (sequence) edit distance, but we see two striking advantages of the watermark concept for barcoding. First, the watermark concept contains an implicit synchronization technique, that does not need preambles or markers to find boundaries of code words within an unknown sequence. Embedding of barcodes in an unknown context is not generalized in approaches considering barcodes based on (sequence) edit distance. A two-ended embedding of sequences is not reflected in this metric. Furthermore, we are able to give an optimal decoding procedure, adapted to a specific error channel. In short, this enables a maximum degree of freedom for existing as well as future experimental settings. Decoding speed is the second important aspect of multiplexing approaches, as the number of barcodes and the available read-length dramatically increased within the last few years. The decoding of barcodes based on watermarks also provides an efficient method for fast decoding of large scale multiplexing approaches.

## Methods

The main concepts presented in this section originates from the ideas first given by Davey and MacKay [20]. The fact, that quaternary watermark codes can be applied for DNA sequencing was preliminary outlined in [23], but sequence constraint on practical oligonucleotides can not be found in this conference paper. Let us denote some basic facts about barcodes first and postpone specific sequence constraints to the section about reasonable encoder settings.

For applying the concepts of watermark codes on DNA barcoding we have to focus on the following constraints, with implications for the modifications we consider here. We highlight our contribution to the basic concepts with the following barcode constraints: A barcode is

- a quaternary sequence. Therefore we will propose generalized non-binary models and concepts here (the original channel was considers strictly binary).
- in general embedded in other sequences. Hence we give an adapted transmission scheme and a novel approach to detect barcode boundaries (in [20] the watermark pattern is repetitive and symbols are decoded as stream).
- length-limited. That consequently restricts the number of code-constructions for which an adaption is practical (long LDPC codes are use in the original paper, not plausible for short barcodes).

We will stress additional contributions to the work of Davey and MacKay, where needed. Let us start with a model for the sequencing process and continue with encoding and decoding based on watermarks.

### Sequencing model

We will first define a communications theoretic model to formally describe the barcoding application.

#### Substitution model

A simplistic communication theoretic model for the sequencing of barcodes has been proposed in [7]. Namely, a fixed barcode word $\mathbf b \in \mathcal A^{N}$, with $\mathcal {A} = \{A,G,C,T\}$ as set of possible symbols (nucleic acids), is entering a *communication channel* with output $\mathbf {r} \in \mathcal A^{N}$, where the channel itself is described by the conditional probabilities to receive a string **y** if **x** was sent, for $\mathbf {x}, \mathbf {y} \in \mathcal A^{N}$.

For our purposes this model has to be extended into two directions: First, a barcode word **b** is assumed to be embedded into a randomly chosen context to obtain the sequence **t** (details will be given below). Second, the sequence **t** is sent over a so-called *Sequencing Channel* resulting in the received word **r**. The channel not only substitutes symbols from **t**, but is also able to delete or insert symbols, hence the length of **r** and **t** may differ.

#### Embedding of barcodes

Given a barcode word **b** of length *N* the sequence **t** is obtained as the concatenation of two random sequences **t**_pre_, **t**_post_ and the sequence **b** as follows

$$\mathbf{t} = \underbrace{t_{1}\dotsb}_{\mathbf t_{\text{pre}} } \underbrace{t_{\delta+1} \dotsb t_{\delta + N}}_{\mathbf b=b_{1}b_{2} \dotsb b_{N}} \underbrace{\dotsb t_{L}}_{\mathbf t_{\text{post}}} \in \mathcal A^{L}, $$

with *δ*∈{0,1,..,*L*−*N*}. The sequences **t**_pre_ and **t**_post_ are assumed to have a random length and the symbols are chosen uniformly at random, hence the length *L* of **t** is a random variable. We will later choose the lengths of the embedding sequences according to a quasi-normal length-distribution on integers (see Section Simulations).

#### Barcoding and working hypotheses

In this paragraph we like to focus on two different paradigm for sequence embedding in our barcoding approach: First we like to address the total embedding of barcodes. Well, for most sequencing scenarios, we find the barcodes next to fixed technical sequences, which are more reliable due to sequence specific (biological) reactions, e.g. primer or adapter oligonucleotides. For sequencing experiments, where we can rely on the exact knowledge of the position of a barcode at one end, highly optimized code words has been proposed in [19]. As an extension of our work is possible to include **t**_pre_ or **t**_post_ as partially known, nevertheless we want to restrict ourselves to have no prior knowledge about the context. The main gain from this very strict premise is a striking freedom of experimental setups for which we can apply our concepts. For example on platforms with paired end sequencing we are able to decode barcodes independent of the direction of reads not restricting the reads to start with a barcode symbol.

The second aspect is the assumption of inherent barcoded sequences. In this manuscript we focus on the problem of decoding (discrimination of code word sequences), conditioned on the fact that a code word is present in the multiplexed sequences. The problem of detecting a barcode (if it is not guaranteed that every sequence contains one) is a more challenging problem, which we want to avoid in the present assay. On codes based on sequence edit distance this extended problem is addressed for, e.g. the PacBio SMRT platform in [24]. We conjecture that the detection problem of barcodes based on watermarks can be solved in future investigations. Such investigation might also lead beyond the barcoding for sequencing applications, which we will address at the end of this manuscript. Nevertheless, we rely on barcoded samples for the following considerations.

#### Sequencing channel model

We define a very simplified model for sequence errors and discuss some aspects of oversimplification in the next paragraph. Let us describe the processes involved in sequencing as a memoryless quaternary channel, i.e. each symbol is handled independently of others. This channel model is specified by a set of parameters **S**, *p*_*i*_ and *p*_*d*_ that are integrated as follows: A transition matrix $\mathbf S \in \ensuremath {\mathbb R}^{4\times 4}$ which describes the substitution probabilities, and *p*_*i*_ and *p*_*d*_ to specify insertion and deletion probabilities. The channel is modeled as an infinite state-machine on symbol level, in which a symbol *t*_*i*_ is queued to pass the channel and therefore will undergo one of the following three events: With probability *p*_*i*_, the symbol *t*_*i*_ remains in the queue and the received stream is prolonged with a random inserted symbols where we assume an uniform distribution on $\mathcal A = \{A,G,C,T\}$. With probability *p*_*d*_, the actual queued symbol is deleted. With probability *p*_*t*_=1−*p*_*i*_−*p*_*d*_ the symbol *t*_*i*_ is passed to a substitution channel which substitutes the symbol *t*_*i*_ according to the transition matrix **S**, with transition probabilities *S*(*r*_*j*_,*t*_*i*_)=*P* *r*(*r*_*j*_|*t*_*i*_). In order to downsize the number of parameters in our model, we will consider **S** as symmetric 4-ary substitution matrix later, i.e., we consider substitutions from a base into another with a single error parameter (similar to the model proposed by Jukes and Cantor [25]). Nevertheless, the model considered here would be able to mimic a refined channel, if exact empirical parameters can be considered.

#### Empirical parameters for a channel model

Empirical data about sequence errors can be seen as the crux of all HMM based approaches in the field of DNA sequencing, as predictions can only perform as good as the underlying assumptions. But, aside from advertising error rate of the big vendors of sequencing devices, real estimates are rather rare to find in literature. Further, there is no real agreement about a common technique to mine such data in a correct way, e.g, using sequence alignment to predict error count is recently in critic of over-fitting, due to predefined alignment costs with impact on the estimates. The commutability of substitutions, insertion and deletion events (a substitution is equal to a deletion followed by an insertion) made things even more difficult. Additionally, there are some indications, that the sequencing channel is more complex as the model we utilize in our approach: Sequencing errors seem to be highly depend on the sequence context, with extended implications on the distribution of symbols in sequenced reads. For Illumina this was shown, e.g. in [26]. We know that the DNA polymerase molecule is prone to bursts of insertions and deletions, if for example repetitive symbols (homopolymers) are present in the physical template sequence. Note, that the reliability of the approach presented here is sensitive to empirical channel parameters to obtain best estimates for demultiplexing samples. A stepwise refinement driven by the feedback of experimental studies is mandatory to adapt watermark barcodes for the demands of different sequencing platforms.

### Demultiplexing problem

**Figure 1:**
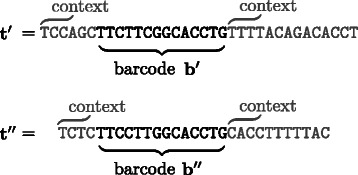
**Sequence embedding of barcodes.** Two example sequences **t** ^′^ and **t** ^″^ labeled with barcodes **b** ^′^ and **b** ^″^ embedded in an unknown DNA context.

During the multiplexing step, different samples are labeled with different barcodes, for example **t** ^′^ is labeled with **b** ^′^ (**b** ^′^ embedded in **t** ^′^) and **t** ^″^ contains **b** ^″^ (see Figure 1). After passing the discussed sequencing channel the resulting sequences **r** ^′^ and **r** ^″^ can differ from **t** ^′^ and **t** ^″^. As the barcodes are possibly affected by errors, **r** ^′^ and **r** ^″^ might be associated to the wrong origin during the demultiplexing step, what is called crosstalk. The encoding and decoding based on watermarks is able to reduce such crosstalk.

### Barcode construction on watermarks

**Figure 2:**
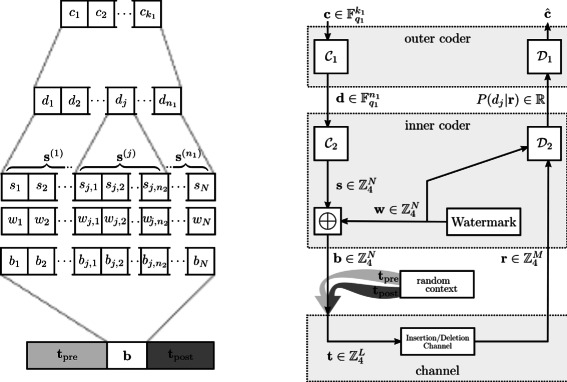
**Transmission scheme for barcodes based on watermark codes.** Consisting of three blocks: 1) outer coder, encoding index words **c** as code words of a linear code $\mathcal C_{1}[\mathbb F_{q_{1}},n_{1}, k_{1}]$ and soft-decoder $\mathcal D_{1}$. 2) inner coder, with inner code $\mathcal C_{2}$ (for sparse sequence representation), decoder $\mathcal D_{2}$ (providing likelihood values) and watermark sequence **w** (known to encoder and decoder). 3) embedding of barcodes in random context and channel with insertion, deletion and substitution errors.

For the construction of barcodes we use concatenated encoding similar to the scheme proposed by Davey and Mackay, which consists of the following two blocks (see Figure 2): An outer code $\mathcal C_{1}$ with parameters $[\!\mathbb F_{q_{1}},n_{1}, k_{1}]$ is a code of length *n*_1_, dimension *k*_1_ and alphabet size *q*_1_ (Galois field $\mathbb F_{q_{1}}$), that provides a set of $q^{k_{1}}_{1}$ code words. Note, that long LDPC outer codes are used in [20]. In order to avoid the constructions of short LDPC codes for barcoding, we will consider different outer codes later. It is worth to mention, that minimal distance and the ability for soft decoding is the only demands on outer codes. We consider information words $\mathbf c \in \mathbb F_{q_{1}}^{k_{1}}$, which are mapped to inner code words $\mathbf d \in \mathcal C_{1} \subset \mathbb F_{q_{1}}^{n_{1}}$. The code $\mathcal C_{1}$ provides a set of code words with a high minimal Hamming distance. The *n*_1_−*k*_1_ redundant symbols are used to arrange outer code words as distant as possible. Such a code can give a code rate of $R_{1}=\tfrac {k_{1}}{n_{1}}$.

The second block consist of an inner encoder, which works in a complete inverse direction. An inner code $\mathcal C_{2}$ is used to create barcode words that have a low Hamming distance to a watermark sequence. The similarity of all barcodes to this watermark pattern is utilized to gain synchronization as explained below. The inner code adds redundancy to the code words by mapping each outer symbols $d_{j}\in \mathbb F_{q_{1}}$ to a sparse sequence representation $ \mathbf s^{(j)} \in \mathbb Z_{4}^{n_{2}}$. The set of sequences **s** ^(*j*)^ with low mean Hamming weight (number of non-zero symbols) can be seen as inner code $\mathcal C_{2}$. The cardinality of this inner code set is *q*_1_ and the rate of the inner code can be stated as $R_{2}=\tfrac {\log _{2} (q_{1}) }{ n_{2} \log _{2} (4) }$. By joining *n*_1_ of these inner code words, a sparse inner block $\mathbf s = \mathbf s^{(1)}\mathbf s^{(2)} \dotsb \mathbf s^{(n_{1})} $ of length *N*=*n*_1_ *n*_2_ is generated.

A barcode word $\mathbf b= \mathbf s \oplus \mathbf w \in \mathcal A^{N}$ is obtained via a symbol-wise *adding* of an arbitrary watermark sequence **w** to the sparse inner block **s** using a fixed mapping of $\mathcal A=\{A,G,C,T\}$ onto $\ensuremath {\mathbb Z}_{4}$, where addition is defined modulo 4 (explicit mappings in Additional file 1 section). The final set of barcodes is denoted as $\mathcal C = \mathcal C_{2} \circ \mathcal C_{1}$ throughout this manuscript. The code  gives a code rate $R=\tfrac {k_{1}}{n_{2}} \tfrac {\log _{2}(q_{1})}{\log _{2}(4)}$.

### Decoding

The decoder consists of two blocks (see Figure 2): An inner decoder $\mathcal D_{2}$, which utilizes a hidden Markov model (HMM) and channel parameters  to provide symbol-wise likelihoods. These are fed into the outer decoder $\mathcal D_{1}$ that performs a maximum likelihood decoding to obtain an estimate $\hat {\mathbf c}$ of the sent code word. We will first define the HMM and explain how this can be used to find the likeliest transmitted sequence (optimal decoding). Afterwards we give a modified HMM, that enables to estimate the boundaries of embedded barcodes and end this section with a suboptimal symbol-wise decoding approach with lowered complexity.

#### HMM for decoding

The basic idea of the HMM presented in this paragraph refers to considerations in [20]. To explain the function of the HMM for decoding it is helpful to ignore the random context and the embedding of code words initially. Therefore we assume **t**_pre_ and **t**_post_ to be absent and the transmitted sequence is exactly one barcode, i.e. **t**=*t*_1_ *t*_2_··*t*_*M*=*N*_=**b**.

If no insertions or deletions occur in a channel the received sequence **r**=*r*_1_ *r*_2_⋯*r*_*L*_ is as long as the sent sequence **t**, i.e. *L*=*M*, but some symbols *r*_*i*_ might differ from *t*_*i*_. Assuming that errors occur independent of the position and equally distributed, we can use fixed substitution probabilities to describe the channel.

For channels with insertion and deletion events the symbol-wise fixed association $t_{i} \leftrightsquigarrow r_{i}$ is usually lost. For example, for a single insertion event at the *i*-th symbol, *t*_*i*_ will be associated to *r*_*i*+1_. A single deletion event before transmitting the *i*-the symbol, will shift *t*_*i*_ to the symbol *r*_*i*−1_ in the received sequence. Obviously, such errors accumulate during the transmission.

One of the main problems of decoding is to estimate the number of insertions and deletions given a received sequence **r**. Therefore we define the *drift* *x*_*i*_ at the *i*-th transmit symbol as (*#* insertions) - (*#* deletions) that occurred in the received sequence before taking *t*_*i*_ into account. The drifts {*x*_*i*_} can be seen as the hidden states of an HMM.

**Figure 3:**
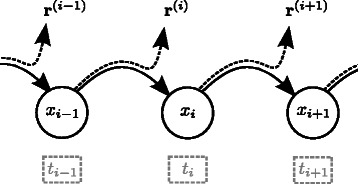
**Illustration of the HMM.** Exemplary series of transitions of the HMM and observables **r** ^(*j*)^, based on state transition *x*_*j*−1_→*x*_*j*_ and transmit symbols *t*_*j*_ for *j*∈{*i*−1,*i*,*i*+1}.

Further, we assume the received sequence

$$\mathbf r =r_{1}r_{2} \cdot\cdot r_{M}= \mathbf r^{(1)}\mathbf r^{(2)} \cdot\cdot \mathbf r^{(L)} $$

to be assembled of sub-sequences **r** ^(*i*)^, as observables, based on the hidden state *x*_*i*_ and the transmit symbol *t*_*i*_. Every state transition *x*_*i*−1_→*x*_*i*_ causes an emission of a sub-sequences **r** ^(*i*)^, that is associated to the position *i* in **t** (in general HMMs the emissions are associated to single states and not to transitions, compare with Figure 3). To characterize the transition probabilities among hidden states and the emission probabilities of observables in the HMM, we use the following set of parameters $\mathcal H:\{ \mathbf S, p_{i}, p_{d}, I \}$. Although we used an identical notation for parameters as before (infinite state-machine in section *Sequencing Channel*), the channel model and the HMM discussed here are not equivalent.

The matrix **S** is parametrizing substitution events, with *p*_*i*_ and *p*_*d*_ we model the probabilities of insertion and deletions and *I* is used to limit the maximal length of observables. For computational reasons we suppose that only *I* consecutive (leading) insertions can be present in observables (the channel model is capable to insert an infinite number of symbols). We further assume that the last symbol of an observable **r** ^(*i*)^ is created by either deleting the actual transmit symbol *t*_*i*_ or appending *t*_*i*_ at the end, substituted according to **S**. If *t*_*i*_ is deleted, we can either just observe **r** ^(*i*)^=*ε* (the empty symbol) or the last symbol of the observable will be a random (inserted) symbol, if leading insertion are present. This limits the set of observables in our model to

$$\mathbf r^{(i)} \in \left\{\epsilon, t_{i}, \overline{t}_{i}, *t_{i}, *\overline{t}_{i}, **t_{i},\dots, \underbrace{** \dotsb *}_{I} \overline{t}_{i} \right\}, $$

where we use wild cards ∗ to symbolize random leading insertions. With $\overline {t}_{i} \in \mathbb Z_{4} \setminus t_{i}$ we denote symbols differing from *t*_*i*_.

Now we give the transition and emission probabilities of the stated HMM. Due to our non-binary adaption, we use a slightly different notations than used in the original approach in [20]. An elementary part for both probabilities is the length distribution of observables, that can be derived from

$$\alpha_{l}(l') = \left\{ \begin{array}{lll} 1 && \text{for}\; l' = 0 \\ \frac{(p_{i})^{l'} }{1-(p_{i})^{I}} && \text{for}\; 0 < l' < I \\ 0 && \text{else}, \end{array}\right. $$

the probability of observing *l* ^′^ random insertions, conditioned on an observable of length *l*. The probability for a sequence **r** ^(*i*)^ of length *l* is given by

$$p(l)=\alpha_{l}(l) p_{d} + \alpha_{l}(l-1) p_{t}, $$

with *p*_*t*_=1−*p*_*i*_−*p*_*d*_. The sequence can be obtained via *l* insertions and deleting *t*_*i*_ or via *l*−1 leading insertions and attaching *t*_*i*_ (or $\overline {t}_{i}$) as last symbol.

The transition probability of the HMM can easily be stated as Pr(*x*_*i*_|*x*_*i*−1_)=*p*(*l*) by choosing *l*=*x*_*i*_−*x*_*i*−1_+1, i.e. two consecutive drift states determine the length of observables and vice versa. The joined probability Pr(**r** ^(*i*)^,*x*_*i*_|*x*_*i*−1_) of observing **r** ^(*i*)^ with a state transition *x*_*i*−1_→*x*_*i*_ is

$$Q^{S} (l,r^{(i)}_{l},t_{i}) = \tfrac{\alpha_{l}(l)}{ 4^{l}} p_{d} + \tfrac{\alpha_{l}(l-1)}{4^{l-1}} p_{t} S (r^{(i)}_{l}, t_{i}), $$

with 0≤*l*≤*I*+1. Explicitly, **r** ^(*i*)^ can consists of *l* random insertions and is not associated with *t*_*i*_ (as *t*_*i*_ is deleted with probability *p*_*d*_). It is also possible to have *l*−1 leading insertions and *t*_*i*_ is substituted according to the substitution matrix **S**. The probability *Q* ^*S*^ can easily be used to calculate likelihoods $\text {Pr}(\mathbf r | \mathcal H, \mathbf t)$ as shown in the next section. Note, for the original binary perspective in [20] no matrix representation of the substitution probability is needed. Here we generalize the basic concepts.

#### Optimal decoding

Given a received sequence **r**=*r*_1_ *r*_2_⋯*r*_*L*_, the set of possible barcodes $\mathbf b = b_{1} b_{2} \dotsb b_{N} \in \mathcal C$ and model parameters  we can describe the decoding as the following maximization approach

$$\hat{\mathbf b} = \arg \max_{\mathbf b \in \mathcal C} \{\text{Pr}(\mathbf r | \mathcal H, \mathbf b) \}. $$

The sequence **r** is most likely to be captured, if $\hat {\mathbf b }$ is assumed to be the transmitted sequence and the channel exactly acts as described with the model parameters . Let us shortly explain how $\text {Pr}(\mathbf r | \mathcal H, \mathbf b)$ can be calculated for the given HMM. The following methods can be found in common text books or, e.g. in [27], but we recall the calculations as we focus on a very special kind of HMM here. Therefore we have to associate the observables of the HMM to received symbols, which is achieved with the drift states. If the barcode symbol *b*_*i*_ is assumed to be the *i*-th transmit symbol *t*_*i*_, then *b*_*i*_ can be associated to the *i*-th observable **r** ^(*i*)^ by the HMM. The drift state *x*_*i*_ can be used to associate the last symbol $r^{(i)}_{l}$ of an observable to the symbol $r_{i+x_{i}}$ in the received sequence **r**. To calculate likelihood values we consider the so-called forward metric

$$F_{i}(y) = \text{Pr}\left(r_{1} \dotsb r_{i-1+y}, x_{i-1}=y | \mathcal H,\mathbf b\right) $$

as probability of having reached the drift state *y* and received symbols *r*_1_··*r*_*i*−1+*y*_ before considering the *i*-th transmitted symbol. In a transmission setting where **t**=**b** we can not have any non-zero drift state before considering the first symbol *b*_1_. Thus we define *F*_1_(*y*)=Pr(*x*_0_=*y*) as *F*_1_(*y*=0)=1 and *F*_1_(*y*≠0)=0. The forward metric can easily be calculated as

$$F_{i + 1}(y) = \sum_{l= 0 }^{I+1} F_{i} (y - \delta_{l}) Q^{S}(l,r_{i+y},b_{i}), $$

with *δ*_*l*_=*l*−1, for 1≤*i*≤*N* and 1≤*i*+*y*≤*M*. The likelihood values $\text {Pr}(\mathbf r | \mathcal H, \mathbf b)$ can be obtained as *F*_*N*+1_(*L*−*M*). Using this optimal decoding approach there is no need for outer decoding and demultiplexing ends up with an estimated barcode as output of the inner decoder. For large sets of code words this maximum likelihood approach becomes computation expensive, as for an outer code of dimension *k*_1_ and symbol alphabet of size *q*_1_ there are $q_{1}^{k_{1}}$ calculations of the forward metric left for decoding. Before we give a suboptimal decoder with reduced complexity, we have to consider how sequence boundaries can be obtained for barcodes embedded in random context.

#### HMM to estimate barcode boundaries

Up to this paragraph we have not discussed the role of the watermark in the transmission. We have just illustrated an HMM able to give the likeliest received sequences **r** conditioned to a hidden transmit sequence **t** and parameters . We will now take a closer look at the watermark and how the inner encoding can be understood from a communication theoretic point of view (see Figure 2).

Recall that a barcode is constructed via addition of two quaternary sequences $\mathbf s, \mathbf w \in \mathbb {Z}_{4}^{N}$ as **b**=**s**⊕**w**. Due to the symmetry of the addition, there are two ways to perceive a data transmission: Apparently there is the transmission of $\mathbf s \overset {\mathbf w}{\to } \mathbf b$ with **w** causing some distortion. A further valid perspective of the transmission is $\mathbf w \overset {\mathbf s}{\to } \mathbf b$, which takes **w** as source with **s** causing substitution errors. Here we stick to the last concept treating the symbols *s*_*i*_ as independent and identically (iid) distributed errors on **w** (which is used to find the boundaries of barcodes).

Given an iid assumption for symbols *s*_*i*_ and the inner code $\mathcal C_{2}$ we can calculate an extended substitution matrix **S** ^′^, with probabilities *S* ^′^(*b*_*i*_,*w*_*i*_) for placing a symbol *b*_*i*_ in the barcode, when a *w*_*i*_ is present at position *i*. We use the matrix **S** ^′^ to define an upstream meta-channel that causes additional substitutions to those generated by the real sequencing channel. In analogy to the previous paragraph, we can state emission probabilities

$$Q^{E}_{w_{i}} \left(l,r^{(i)}_{l}\right) = \tfrac{\alpha_{l}(l)}{ 4^{l}} p_{d} + \tfrac{\alpha_{l}(l-1)}{4^{l-1}} p_{t} E\left(r^{(i)}_{l},w_{i}\right), $$

for observing a certain **r** ^(*i*)^ if the symbol *w*_*i*_ is present at position *i*. With

$$E\left(r^{(i)}_{l},w_{i}\right)= \sum_{b} S\left(r^{(i)}_{l},b\right) S'\left(b,w_{i}\right) $$

we denote the *effective* probability of having substitutions due to the sequences **s** and the substitution matrix **S**. The task of estimating barcode boundaries can be reduced to the estimation of the likeliest sequence of drift states $\text {Pr}(x_{1}x_{2} \cdot \cdot x_{N+1} | \mathcal H, \mathbf r, \mathbf w)$ in the HMM using $Q^{E}_{w_{i}}$ as we show in the next section.

#### Finding barcode boundaries

For embedded code words we can understand the symbol *b*_*i*_ as shifted transmit symbol *t*_*δ*+*i*_ and thus *b*_*i*_ has to be linked to the observable **r** ^(*δ*+*i*)^ in the HMM (see section *Embedding of barcodes*). But we can easily integrate the sequence offset *δ* as initial drift *x*_0_. For the symbols *b*_*i*_ we therefore redefine the drift states {*x*_*i*_} for the HMMs according to embedded symbols.

To calculate likelihood values for received sequences, we now consider the forward metric

$$F_{i}(y) = \text{Pr}(r_{1} \dotsb r_{i-1+y}, x_{i-1}=y | \mathcal H,\mathbf w) $$

as probability of having reached the drift state *y* and received symbols *r*_1_··*r*_*i*−1+*y*_ before considering the *i*-th watermark symbol. We furthermore have to determine an initial distribution for the quantity *F*_1_(*y*) to be able to calculated the forward metric

$$F_{i + 1}(y) = \sum_{l= 0 }^{I+1} F_{i} (y-\delta_{l}) Q^{E}_{w_{i}} \left(l,r_{i+y} \right), $$

with *δ*_*l*_=*l*−1. We can furthermore calculate backward quantities

$$B_{i}(y) = \text{Pr}(r_{i+1+y} \dotsb | x_{i}=y, \mathcal H,\mathbf w) $$

as probability of receiving a certain tail of symbols starting with *r*_*i*+1+*y*_ given a state *y* associated with the *i*-th watermark symbol, which can be calculated as

$$B_{i - 1}(y) = \sum_{l= 0 }^{I+1} B_{i} (y+\delta_{l}) Q^{E}_{w_{i}} \left(l,r_{i+y+\delta_{l}} \right). $$

If we have a good guess for the distribution *F*_1_(*y*_1_) and *B*_*N*_(*y*_*N*_), i.e. an a priori distribution of having barcode boundaries at position *y*_1_ respective *N*+*y*_*N*_, we make use of it. To enable an alignment of the embedded barcodes, we have to introduce novel prior distributions, slightly different to those proposed by Davey and MacKay. Anyway, a conservative approach is setting non-zero probabilities for $F_{1}(y) = \tfrac {1}{M}$, where 0≤*y*≤*M* and *B*_*N*_(*y*)=1 on drift positions −*N*≤*y*≤*M*−*N*. Here we have to differ from the original concept [20], which is needed to detect a single non-repetitive watermark within an unknown context.

The likeliest drift associated with the *i*-th embedded symbol is finally inferred as

$$\hat{x}_{i} = \arg \max_{y} \left\{\text{Pr}(y | \mathcal H, \mathbf r, \mathbf w) \propto F_{i+1}(y) B_{i}(y) \right\}. $$

The estimates $\hat {x}_{0}$ and $\hat {x}_{N}$ are used to refer to the barcode boundaries in the received sequence.

#### Symbol-wise likelihoods

We can finally perform a symbol-wise decoding as follows: The forward and backward metric does not only provide estimates for the start and the end position of an entire barcode word, but also enables to calculate conditional likelihoods

$$P\left(\mathbf r |\mathcal H,\mathbf w, \mathbf s^{(j)} \right)= \sum_{y,z} F_{u}(y) \pi_{u}\left(y,z,\mathbf s^{(j)}\right) B_{v}(z) $$

based on inner code words $\mathbf s^{(j)} \in \mathcal C_{2}$. With the indexes *u*=(*j*−1)*n*_2_+1 and *v*=*j* *n*_2_ we denote delimiters of **s** ^(*j*)^ (compare Figure 2). The drifts *y* and *z* define potential boundaries *u*+*y* and *v*+*z* of an emitted sub-sequence of **r** that is assumed to depend on **s** ^(*j*)^. With *π*_*u*_(*y*,*z*,**s** ^(*j*)^) we symbolize a truncated version of the forward metric, starting at states *y* and ending at states *z*. For the evaluation of *π* we further consider emission probabilities $Q^{S} \left (l,r^{(i)}_{l},w_{i} \oplus s_{i} \right)$. As the inner code words are determined by outer code symbols, i.e. $\mathcal C_{2}: d_{j} \to \mathbf s^{(j)}$, we can easily derive symbol-wise marginal a posteriori probabilities $P(d_{j}| \mathcal H, \mathbf r)$ from the conditional likelihoods. The symbol-wise marginals are finally utilized in the outer coder (see Figure 2).

Using this suboptimal inner decoding approach, we are able to decrease the computational costs to *q*_1_ *n*_1_ evaluations of the forward metric *π* (compare section *Optimal decoding*). As we need an additional soft decoding for the outer code, there are further operations needed: For a maximum likelihood approach we have to consider ${q_{1}^{k}}$ code words and search for the likeliest solution.

### Simulations

In order to perform an in silico application of barcodes based on the watermark concept, we first have to define some reasonable setting for encoding, which is already quite challenging.

#### Reasonable encoder settings

As stated before (see section *Barcode construction on watermark*), there are different parameters, which influences the concepts and for which we have to find a reasonable setup to run simulations. First there is an outer codes, which should in combination with the inner coding lead to short barcode words, because we do not want to produce exceptional overhead with multiplexing (tagging) target fragments. There is the minimum distance of outer code words and the sparsity of the inner encoder, which can independently be characterized. And finally we have the watermark sequence, which can randomly be chosen. We utilize the degree of freedom with the watermark to incorporate with additional sequence constraint, that barcodes should fulfill to be experimentally valid. Therefore we run a greedy search for the watermark pattern that maximizes the number of barcodes that meet all sequence constraint. But let us consider all particular setting one by one in the following paragraphs.

#### Suitable outer codes

For the construction of barcodes we focus on a target-length of *N*=*n*_1_ *n*_2_∈[12,..,25] symbols with *n*_1_∈{3,4,5,6} and *n*_2_∈{4,5,6,7,8}. Further we limit the outer code $\mathcal C_{1}\left [\mathbb F_{q_{1}},n_{1},k_{1},\text {d}_{H}\right ]$ to the best known linear codes listed in [28] for several cardinalities of Galois fields $\mathbb F_{q_{1}}$, for which we considered *q*_1_∈{2,3,4,5,7,8,9}. Long LDPC codes has been used in the original approach of Davey and MacKay (see [20]), but as the construction of short LDPC codes would be somehow confusing for readers involved in channel coding, we decided to use the best known linear codes. But we might note, that the hamming distance and the ability for soft decoding is the only demand on outer codes here and LDPC codes are likely to perform equivalent. The minimal Hamming distance d_*H*_ of the best known linear codes we used is either maximal or the highest known regarding given parameters. Additionally we bound the dimension *k*_1_ to achieve code set sizes $48 < |\mathcal C_{1}|=q_{1}^{k_{1}} < 1000$. This guarantees a certain minimal code rate on one side and limit the computational effort of the outer decoding the other side. We end up at 263 possible code configurations, but most of the resulting codes perform disastrous with the watermark concept, because the watermark is heavily corrupted by inner code words.

#### Sparsity of the inner code

We consider the density (mean Hamming weight) as

$$\overline{\text{w}}_{H}(\mathcal C_{2}) = \frac{1}{\text{n}(\mathcal C_{2}) |\mathcal C_{2}|} \sum_{\mathbf s \in \mathcal C_{2}} \text{w}_{H}(\mathbf s) $$

for the inner code, with w_*H*_(·) as Hamming weight, n(·) as length and |·| as cardinality of the code. The inner code $\mathcal C_{2}$ needs to exhibit a low density, to keep the watermark shining through the barcodes, when inner code words are added. For large densities there is no ability left to detect the barcode boundaries and consequently decoding will fail completely. Inspired by the approach in [29] we avoid the all-zeros code word in $\mathcal C_{2}$, but further bound the density to $\overline {\text {w}}_{H}(\mathcal C_{2}) < 0.3 $, to keep the watermark present. Finally, we end up in a set of 73 parameter configurations for *q*_1_,*k*_1_,*n*_1_ and *n*_2_. For each of the 73 different parameter sets we run a brute-force search (10^7^ trials), where we iteratively selected one inner code and watermark randomly to produce barcode sets. From the evaluated settings we kept the one, which met the following sequence constraints.

#### Sequence constraints

We filtered for code words with unbalanced counts of symbols, to respect limitations on the *GC content* of barcodes. Such filtering can be seen as a de facto standard in the construction of barcodes (see for example [1,7,19]) and is related to technical constraints due to the preparation and the sequencing of genomic material. The relative frequency of a subset of two symbols should not be below 40% and above 60% in each barcode, otherwise we excluded the barcode. We furthermore exclude barcodes with *prefect self-complementation* and more than two *sequential repetitions* of the same base (homopolymer length), similar to the restrictions stated in [24]. We consider this settings as sufficient and strict enough to avoid experimental problems during the preparation in real sequencing tasks. Discarding such inappropriate code words means an additional loss in code rate.

#### Increasing the mean edit distance

For decoding based on the HMM approach, edit distance implicitly matters, thus we try to increase the mean edit distance of code word with a simple strategy. For two sets of barcodes with identical counts of remaining barcodes (after filtering) we keep the one maximizing the mean edit distance

$$\overline{\text{d}}_{E}(\mathcal C) = \frac{1}{ \text{n}(\mathcal C)(\text{n}(\mathcal C)-1)} \sum_{\mathbf b_{i}, \mathbf b_{j} \in \mathcal C: i\neq j} \text{d}_{E}(\mathbf b_{1},\mathbf b_{2}), $$

where d_*E*_(**b**_1_,**b**_2_) denotes the edit distance [30] of barcodes **b**_1_ and **b**_2_, which can be understood as the number of single-symbol sequence operations to transform **b**_1_ into **b**_2_ or vice versa. But, as the edit distance is bounded by the hamming distance, we do not gain a lot with this final heuristic refinement step.

#### Estimating the decoding error

To evaluate the refined set of 73 codes, we give the following demultiplexing scenario. For each code we consider an error curve according the estimates of the decoding error probability on different channel settings. The estimation of a single point in the error curve is based on a set of 200,000 barcodes, which we refer to as batch. Each batch is processed with a certain symbol mutation probability *p*_*mut*_∈[.01,.16]. This value determines a symbol-wise probability for an edit operation that can be caused by our channel model. Similar approach has been considered in [19], but we like to be a bit more precise with the description of the modifications of the channel model. Buschmann et al. [19] considered a minimal mutation probability of 10^−1^ for their evaluation, but others claim, that error rates are several orders lower [11]. We will take such indications into account.

Each barcode from a batch is embedded in a random context of variable length (compare section *Embedding of barcodes*). We use normal distributed random variables (with mean *μ*_*l*_=50 and variance *σ*_*l*_=5) to determine the length of **t**_pre_ as well as **t**_post_. We simply take the nearest non-negative integer to define the length of the random context, which is uniformly distributed on {*A*,*C*,*G*,*T*}.

We further use a state machine to produce erroneous received sequences based on the following four events: correct transmission C, substitution S, insertion I and deletion D. In slightly different notation to the former model (see. *Sequencing Channel*) we assign probabilities to the events C and S and do not use conditional probability like a substitution matrix **S**.

The probability for correct transmission C or substitution S is equal to *p*_*t*_ in the former representation. The probability for C equals 1−*p*_*mut*_ and the probability for any of the error events (S,I or D) is *p*_*mut*_. Every error event S, I or D is equal likely. To obtain an equal distribution among the mentioned events, it is easier to use the present notation, but equivalent behavior can be approached with both versions of the channel state-machines.

To save decoding time we iteratively pass each transmit sequence through the state machine, until we have at least one error event within the barcode region. The probability of having a defective code word of length *N* is Pr(def)=1−(1−*p*_*mut*_)^*N*^. We further considered an error-free barcode to be decoded perfectly, i.e. $\text {Pr}(\texttt {err} | \overline {\texttt {def}})=0$, with err denoting the event of decoding error. Please note that this oversimplifies the false positive rate introduced by sporadic similarities of the context with dedicated barcode words. The rate is supposed to grow linear with the context length and the size of barcodes set. Nevertheless, the probability for false positive events exponentially tend to zeros with the length *N* of the code words. For barcodes of length ≥12 embedded in 100 random symbols we consider this error as marginal offset for the estimated decoding errors. Our evaluation finally end up in estimating the conditional error Pr(err|def), which gives an estimate for the unconditioned error probability as *P*_*e*_=Pr(err|def)Pr(def).

## Results and discussion

First we give a rough overview on meaningful properties of watermark-based barcodes with the considered 73 parameterizations. Furthermore, we provide a refined analysis and evaluation for certain codes in the already defined decoding scenario. To minimize the textual elements in the figures, we use the following notation: *q*_1_|*k*_1_|*n*_1_|*n*_2_ indicates the concatenated coding using an outer code $\mathcal C_{1}\left [\mathbb F_{q_{1}},n_{1},k_{1}\right ]$ and an inner code $\mathcal C_{2}: \mathbb F_{q_{1}} \to \mathbb Z_{4}^{n_{2}}$. The particular codes can be found in the Additional file 1 section of this paper.

### Properties of barcodes based on watermarks

**Figure 4:**
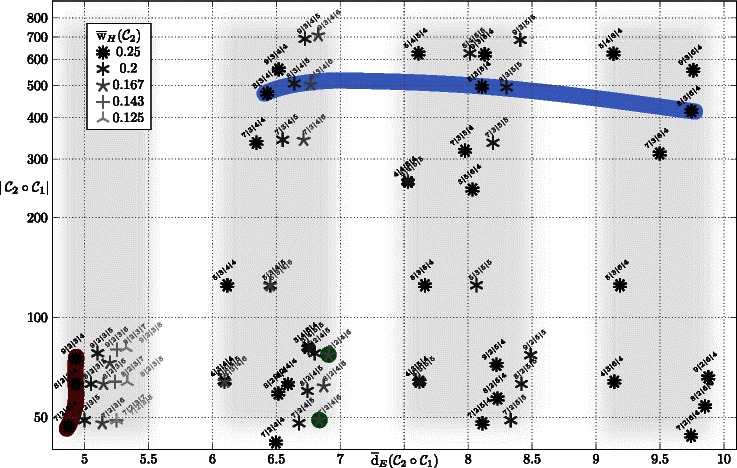
**Properties of the examined barcodes based on watermarks.** Star-symbols indicate the mean density and two dimensional coordinates link the mean edit distance $\overline {\text {d}}_{E}$ and the size $|\mathcal C_{2} \circ \mathcal C_{1}|$ of codes. Parameters of the barcodes are labeled as *q*_1_|*k*_1_|*n*_1_|*n*_2_, denoting an outer code $\mathcal C_{1}\left [\mathbb F_{q_{1}},n_{1},k_{1}\right ]$ and inner code $\mathcal C_{2}: \mathbb F_{q_{1}} \to \mathbb Z_{4}^{n_{2}}$. Codes evaluated in refined analysis/simulations (see. Figure 5 and 6) are colored.

In Figure 4 we link the principal characteristics as mean edit distance $\overline {\text {d}}_{E}$, mean density $\overline {\text {w}}_{H}$ and the cardinality $|\mathcal C_{2} \circ \mathcal C_{1}|$ of barcodes in a comprehensive illustration. We use star-symbols to indicated the mean density (several levels) and two dimensional coordinates to link mean edit distance and the size of code sets.

There are distinct blocks, where the influence of the inner code can be separately examined. With fixing the outer code, e.g. *q*_1_|2|3|*n*_2_ or *q*_1_|3|4|*n*_2_ for *q*_1_∈{7,8,9} and increasing the length *n*_2_ of inner code words, one can deduce how code rate is exchanged for lowered mean density and increased mean distance. However, we see configurations, for example 4|3|4|*n*_2_ for *n*_2_∈{4,5,6}, where we have not been able to increase the mean distance by prolonging the inner code. We have observed several clusters, where similar effects can be found.

For the codes *q*_1_|*k*_1_|4|*n*_2_ or *q*_1_|*k*_1_|5|*n*_2_ and *q*_1_|*k*_1_|6|*n*_2_ the effect of the outer code can be separated. We find clusters of star symbols at different mean distances (see darkened areas in Figure 4). These levels can be explained through the different minimum Hamming distance of the outer codes. We have Hamming distances 2,3 and 4 for the present outer codes at *n*_1_ equals to 4,5 and 6. For concatenated coding it is known that the minimum distances of inner and outer codes are multiplicative [31]. As the edit metric is upper bounded by the Hamming distance, we anticipate the described levels for edit distances. The mentioned leveling can consistently be found for all outer codes.

**Figure 5:**
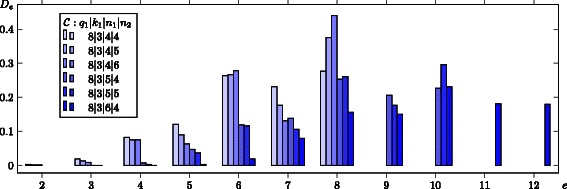
**Exemplary distance distribution of a sets of barcode based on watermarks.** *D*_*e*_ denotes the relative number of code-pairs with an edit distance equal to *e*. Each barcode set consists of maximal 512 code words, according to the examined outer codes $\mathcal C_{1}\left [\mathbb F_{8},n_{1},3\right ]$ (see also Figure 4, in blue). The distance distribution is normalized regarding the cardinality of code words after filtering.

Despite we have maximized the edit distance of barcodes on average, it is also interesting to focus on the pairwise distance of barcodes. To examine the distance in detail we utilize the so-called distance distribution. In [32] the average number of code words at a certain distance to a fixed code word is considered as an useful distance measure for non-linear codes based on Hamming metric. The edit distance distribution of a codes  consists of the numbers

$$D_{e} = \frac{1}{M} \big | \!\left\{ (i,j) : \text{d}_{E}(\mathbf b_{i},\mathbf b_{j})= e, \quad \mathbf b_{i},\mathbf b_{j} \in \mathcal C) \right\}\!\big |, $$

where $M = |\mathcal C|(|\mathcal C|-1)$ and d_*E*_ denotes the edit distance. In Figure 5 we illustrate the distance distribution of barcodes with parameters 8|3|*n*_1_|*n*_2_ (Figure 4, in blue). Apparently, there are particular pairs of code words with very low edit distances, but as we recall the code construction based on inner code words with very low Hamming weight, this fact is not too surprising. Nevertheless, some longer codes show a negligible amount of such code words with small edit distances. For instance, in the code 8|3|6|4 less than 1*%* of all possible pairs of barcodes show an edit distance d_*E*_<6.

According to the very strict filtering (see *Sequence constraints*), we had to prune out the sets of $q_{1}^{k_{1}}$ outer code words, what additional lowers the code rate. A detailed description about the excluded barcodes can be found in the Additional file 1.

### Evaluation of decoding

**Figure 6:**
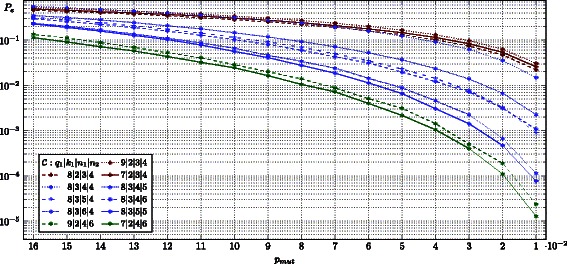
**Simulation results for a realistic decoding scenario.** Estimated decoding error probability *P*_*e*_ for barcode sets with parameters *q*_1_|*k*_1_|*n*_1_|*n*_2_, consisting of an outer codes $\mathcal C_{1}\left [\mathbb F_{q_{1}},n_{1},k_{1}\right ]$ and inner code $\mathcal C_{2}: \mathbb F_{q_{1}} \to \mathbb Z_{4}^{n_{2}}$. All evaluated codes are highlighted in Figure 4 with identical colors. Each barcode set is tested for different symbol mutation probabilities *p*_*mut*_∈[.01,.16]. On average, each randomly drawn barcode is embedded in 100 random symbols before decoding.

In Figure 6 we illustrate the estimates *P*_*e*_ for the decoding error probability of different codes settings. We ran simulations for all 73 barcode set and ranked the sets according the decoding behavior. To give a rough outline for the performance of our approach we show the barcodes, which performed best (Figure 4 and 6 in green) and worst (Figure 4 and 6 in red). A barcode length of 12 (codes *q*_1_|*k*_1_|3|4) seems to be insufficient to provide a good synchronization based on watermarks and thus the majority of decoding errors were found to be caused by synchronization issues (results not shown). The best performing sets of barcodes surprisingly have not occupied the maximal possible length, but 24 symbols. As there are only 49 sequences available, this set of code words provides a poor code rate of $\tfrac {\log _{2}(7^{2})}{\log _{2}(4^{24})} = 0.117$.

A reasonable trade-off between error-correcting capability and cardinality is provided for example by codes with parameters 8|3|*n*_1_|*n*_2_ (Figures 4, 5 and 6, in blue). Although we are facing relative low code rates (compared to approaches like [19]) ranging from 0.188 to 0.281, more than 400 sequences available with quite surprising decoding capability.

### Decoding complexity

According to [20] we utilized a fast decoding approach with reduced complexity for our simulations. We bounded the maximal number of possible drift states {*x*_*i*_} in all HMMs to *Δ*∈{5,..,20} according to the suggestion given by Davey and Mackay. The complexity for decoding a single embedded barcode is $\mathcal O(MLI + q_{1} N \Delta I + q_{1}^{k_{1}})$, with *N* denoting the length of the barcode, that is embedded in a received sequence of length *M*. Decoding is based on the assumption that the channel can introduce maximal *I* inserted symbols and we consider the maximal drift between received and transmitted sequence to be limited by *Δ*. An order of *MLI* calculation are needed to estimate barcode boundaries, *n*_2_ *Δ* *I* operations are needed to provide soft-values for each of the *q*_1_ *n*_1_ possible outer code words (result in *q*_1_ *N* *Δ* *I*) and $q_{1}^{k_{1}}$ final comparisons has to be spent for soft decoding the outer code in the most expensive case (maximum likelihood).

The prototype decoder with which we ran our simulations is implemented in MATLAB. We further parallelized the decoding procedure and created jobs of 10^6^ received sequences, that were processed by single cores (Opteron, 2.6 GHz). The average length of received sequences was in a range of 112 and 150 symbols, resulting in an average processing time of 6 hours for the tasks with lowest calculation costs (code parameters 7|2|3|4). The longest time we needed to complete demultiplexing of 10^6^ sequences (code parameters 9|3|5|5) was strictly below 24 hours (on a single core).

### Future directions

Apart from the theoretical considerations we have given in this manuscript, there are lot of future direction starting from this initial point. Some of them are mandatory to enable an application in real biological experiments, others are modifications of the concepts for extended applications.

Let us first address the essential steps needed to establish an HMM based decoding in real experiments: The HMM, as core of the decoding system, is the most sensible part of the concept. It is mandatory to run experiments to gather reliable data about all channels, the concepts should be used for. From our point of view there is a lack of reliable data about insertions, deletions and substitution errors for possible channel models. For the sequencing application we assume that different platforms show a variety of *sequencing channels*, additionally affected by experimental parameters, e.g. the extend of PCR pre-processing of samples. To obtain an optimal suited decoder, the HMM should be adapted to the considered channels. As most of the channels show a correlation of errors, more complex HMMs should be considered, reflecting a channel model with memory. Finally, it might be possible to establish a self-adaptive algorithm to parametrize the HMMs without any prior knowledge about the ratio of errors in the channel. A suitable statistic and refined calibration steps should be invented. Another important point for estimating the error characteristics is the *construction of watermark codes*. Exact empirical parameters of the channel could be incorporated in the design of watermark codes to improve decoding steps, suited for special channels.

Further aspects that could be considered with the given concepts are the following: Aside from the synchronization aspect in this manuscript, it seems very promising to use the maximum likelihood decoding method for other sequences than watermark codes. Conditioned on good empirical parameters for an underlying HMM one could consider a reliable detection of barcodes based on the *Sequence-Levenshtein distance* in a probabilistic way rather than based on sequence alignment.

In the presented approach we focused on the discrimination of code words, assuming codes are always present in inspected sequences. The detection of code words within DNA context is another big issue that should be solved for future investigations using an HMM based decoding. Resent research shows that even for sequencing approaches the detection of barcodes is quite challenging. In [24] they focus on a specific problem with certain setups on the PacBio SMRT platform. Caused by technical reasons, sporadically barcodes are not present in the sequence data. Another interesting field of application of an HMM based sequence detection could be clonal studies, where the sequenced genome could or could not contain a predefined sequence, which was introduced in ancestor organisms.

## Conclusion

We proposed an adaption of the watermark concept of [20] for DNA barcoding. A generalized channel model for sequencing and suitable modifications of the decoder were defined. Moreover, we investigated in a strategy to choose watermark sequences and inner codes in a reasonable way to enable barcoding in line with common experimental requirements. We provide a code construction, considering the best known linear codes as outer codes and biological sequence constraints to filter for suitable code words, resulting in an exemplary set of 73 different code sets ranging from 12 to 24 nucleotides. The codes are illustrated in a comprehensive scheme, highlighting watermark specific parameters as well as the mean edit distance, to give an impression how watermark based barcodes could be characterized. For a reduced set of codes we finally evaluated the demultiplexing of sequences in a realistic simulation scenario. Within this in silico evaluation we could show that barcodes based on watermarks can theoretically be used for multiplexing. It is remarkable, that even with very short watermark patterns we are able to reliably find the barcodes boundaries in order to discriminate different code words with an HMM based decoder. The probability of decoding errors, which finally leads to the undesired cross-talk phenomenon was found to be very low. Other approaches that investigate barcodes with large (sequence) edit distance [16,19] show significant higher code rates for shorter barcodes, but we have given an entirely different concept that allows for large scale multiplexing approaches, also able to handle insertion and deletion errors.

Moreover, we can provide the marker-less synchronization based on watermarks, to recover the barcode boundaries. This synchronization concept provides an ultimate degree of freedom for experimental sequencing setups as well as for future applications, also apart from the sequencing context.

## Additional file

Additional file 1
**We provide 73 files containing all the examined barcodes.** Further informations are given, like: The watermark in decimal and nucleic acid notation, as well as the mapping of the inner encoder and the used mapping from integers to nucleic acid $\mathbb Z_{4} \to \{A,G,C,T\}$ are given in a preamble. We furthermore give the distance distribution of barcode words. Finally, all barcodes are listed (plus explicit construction via code concatenation). Dropped barcodes, which do not meet the sequence constraints are explicitly indicated.
